# Boosting Power Density of Proton Exchange Membrane Fuel Cell Using Artificial Intelligence and Optimization Algorithms

**DOI:** 10.3390/membranes13100817

**Published:** 2023-09-28

**Authors:** Rania M. Ghoniem, Tabbi Wilberforce, Hegazy Rezk, Samer As’ad, Ali Alahmer

**Affiliations:** 1 Department of Information Technology, College of Computer and Information Sciences, Princess Nourah bint Abdulrahman University, Riyadh 11671, Saudi Arabia; rmghoniem@pnu.edu.sa; 2 Department of Engineering, Faculty of Natural, Mathematical & Engineering Sciences, King’s College London, London WC2R 2LS, UK; tabbi.wilberforce@kcl.ac.uk; 3 Department of Electrical Engineering, College of Engineering in Wadi Alddawasir, Prince Sattam bin Abdulaziz University, Riyadh 11942, Saudi Arabia; hr.hussien@psau.edu.sa; 4 Department of Electrical Engineering, Faculty of Engineering, Minia University, Elminia 61519, Egypt; 5 Renewable Energy Engineering Department, Faculty of Engineering, Middle East University, Amman 11831, Jordan; sasad@meu.edu.jo; 6 Department of Mechanical Engineering, Tuskegee University, Tuskegee, AL 36088, USA; 7 Department of Mechanical Engineering, Faculty of Engineering, Tafila Technical University, Tafila 66110, Jordan

**Keywords:** PEM fuel cell, fuzzy modeling, ANFIS, power density, Salp swarm algorithm, root mean square error, particle swarm optimization, evolutionary optimization, grey wolf optimizer

## Abstract

The adoption of Proton Exchange Membrane (PEM) fuel cells (FCs) is of great significance in diverse industries, as they provide high efficiency and environmental advantages, enabling the transition to sustainable and clean energy solutions. This study aims to enhance the output power of PEM-FCs by employing the Adaptive Neuro-Fuzzy Inference System (ANFIS) and modern optimization algorithms. Initially, an ANFIS model is developed based on empirical data to simulate the output power density of the PEM-FC, considering factors such as pressure, relative humidity, and membrane compression. The Salp swarm algorithm (SSA) is subsequently utilized to determine the optimal values of the input control parameters. The three input control parameters of the PEM-FC are treated as decision variables during the optimization process, with the objective to maximize the output power density. During the modeling phase, the training and testing data exhibit root mean square error (RMSE) values of 0.0003 and 24.5, respectively. The coefficient of determination values for training and testing are 1.0 and 0.9598, respectively, indicating the successfulness of the modeling process. The reliability of SSA is further validated by comparing its outcomes with those obtained from particle swarm optimization (PSO), evolutionary optimization (EO), and grey wolf optimizer (GWO). Among these methods, SSA achieves the highest average power density of 716.63 mW/cm^2^, followed by GWO at 709.95 mW/cm^2^. The lowest average power density of 695.27 mW/cm^2^ is obtained using PSO.

## 1. Introduction

**Figure 1:**
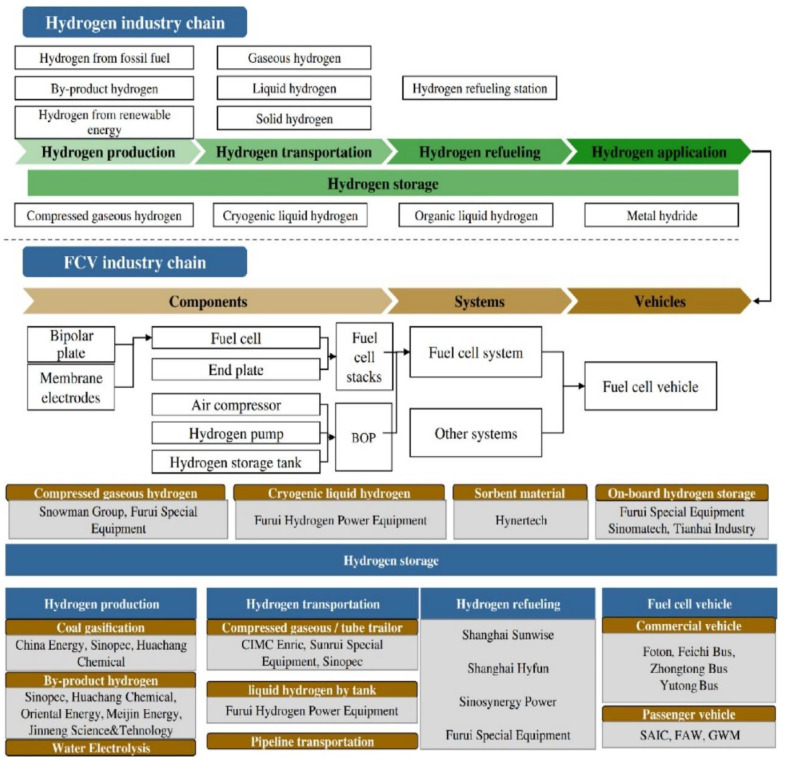
Summary of hydrogen production and application (permission to reproduce [14], License number: 5527071393548).

The world is facing an urgent and critical need to transition away from fossil-based energy sources. This pressing concern is primarily motivated by the harmful environmental consequences associated with fossil fuels and the alarming rate at which their reserves are being depleted [1]. Furthermore, there are other crucial factors that reinforce the need to transition to clean energy sources. Firstly, the prices of fossil fuels are subject to fluctuations, making them economically unstable. Additionally, regions where these resources are extracted often experience political tensions and conflicts, posing ongoing risks and instability. In contrast, embracing clean energy solutions not only provides a more sustainable and stable path forward, but also plays a crucial role in significantly mitigating greenhouse gas emissions, which is essential for addressing climate change and ensuring a sustainable environment for future generations [2,3]. However, it is essential to consider the distinctive challenges associated with renewable energy sources such as wind and solar power. These sources exhibit intermittent generation patterns due to their dependence on weather conditions, resulting in temporal and spatial disparities between energy generation and end-user consumption [4,5]. This intermittent and unstable nature presents significant obstacles in maintaining a dependable and stable power system. In contrast to renewable energy sources, which require energy storage technologies to bridge these gaps and ensure a continuous energy supply, proton exchange membrane fuel cell (PEM-FC) technology based on hydrogen energy boasts a distinct advantage. As long as there is an adequate hydrogen source and a continuous air supply, PEM-FCs can continuously produce electricity [6]. This distinctive attribute makes hydrogen energy an appealing option for achieving a stable and efficient power supply, particularly in scenarios where uninterrupted power generation is critical. Furthermore, hydrogen is widely recognized as an ideal clean energy source for power generation due to its high calorific value and lower carbon emissions. As a result, there is increasing interest and focus on hydrogen FC technologies within the current energy landscape [7]. The United States is currently actively promoting and advocating for the adoption of hydrogen FC technology, primarily due to its potential to significantly improve air quality and address the urgent need to decarbonize the automotive sector coupled with the intermittency associated with renewable energy solutions such as wind and solar. The Made in China 2025 strategy, as outlined in reference [8], aims to achieve a dual objective in China: improving air quality in urban areas and sustaining economic growth. Countries such as Japan are placing a particular emphasis on energy security by focusing on improving the efficiency of hydrogen systems as an alternative to conventional energy-harnessing methods. This approach aims to ensure a stable and reliable energy supply while minimizing the negative environmental impact [9]. According to Habib et al. [10], the transition towards a hydrogen economy is outlined in three key steps. The initial step involves the production of hydrogen from sustainable sources alongside the development of FCs for residential applications. Subsequently, further advancements in FC technology are crucial to enable the integration of FC electric vehicles into the transportation sector. The second step focuses on integrating hydrogen supply chains within the broader energy system by the year 2030. Finally, the ultimate objective is to establish carbon-neutral methods of hydrogen production by 2040. Europe places significant emphasis on the utilization of hydrogen in both industrial and transportation strategies, underscoring its pivotal role in achieving sustainable energy goals [11]. Wang et al. [12] highlighted that the transport sector in the United States accounts for approximately 37% of the total energy consumption. Therefore, the application of PEM-FCs in the automotive sector can significantly reduce energy consumption with minimal impact on the environment. In the Gulf region, countries such as the United Arabian Emirates (UAE) and the Kingdom of Saudi Arabia are increasingly considering investments in vehicle electrification. They are even categorizing hybrid vehicles based on their energy sources, such as battery, FC, etc. [13]. This indicates a growing interest in alternative energy technologies in the region. Figure 1 illustrates the correlation between the hydrogen industry chain and hydrogen FCs in the automotive sector. The hydrogen industry chain encompasses hydrogen production, transportation, refueling, and usage. On the other hand, the FC vehicle industry chain focuses on achieving high FC performance, including individual cell components. These considerations highlight the importance of developing an efficient and integrated hydrogen infrastructure, as well as advancing FC technology, to drive the growth of hydrogen-powered vehicles in the automotive industry.

### 1.1. Principles and Literature Review of Proton Exchange Membrane Fuel Cells (PEM-FCs) 

FCs have emerged as highly recommended energy conversion devices due to their numerous advantages. FCs have emerged as highly recommended energy conversion devices due to their numerous advantages. These advantages encompass a notably higher efficiency, typically ranging from 40% to 60%, in contrast to the efficiency of internal combustion engines, which generally falls between 20% and 35%. Additionally, FCs boast excellent energy density and are renowned for their high reliability. Furthermore, they produce minimal noise since they lack moving parts, making them environmentally friendly [15,16]. However, it is imperative to clarify that when assessing the efficiency of PEM-FCs, it becomes evident that their efficiency is indeed lower than that of lithium batteries, which often exceeds 90% [17].

**Figure 2:**
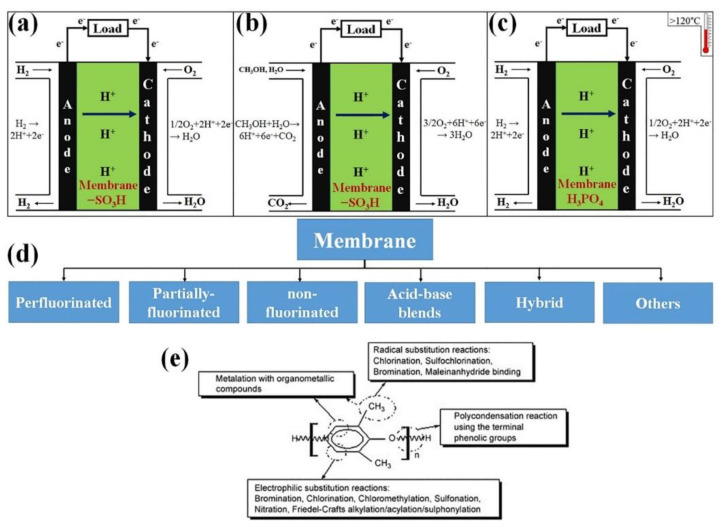
Diagram of (**a**) low temperature PEM-FC, (**b**) direct methanol FC, (**c**) high temperature PEM FC, (**d**) types of membrane for FCs, (**e**) chemical structure of membranes (permission to reproduce [22], License number: 5527130868865).

In recent years, research efforts have focused on enhancing FC performance for various applications. FCs operate through the conversion of chemical energy into electrical energy, resulting in the production of heat and water as by-products of the reaction [18,19]. Basically, FCs harness electric power from the chemical energy stored in a fuel. As long as there is a constant supply of fuel and oxidant, FCs will continue to generate electricity. A typical FC consists of a membrane, an anodic electrode, and a cathodic electrode [20,21]. Figure 2 provides a diagram illustrating the various layers within PEM-FCs. The PEM-FC is among the most recommended FC types for the automotive industry due to its fast start-up times, compact size, and suitability for mobile applications.

**Figure 3:**
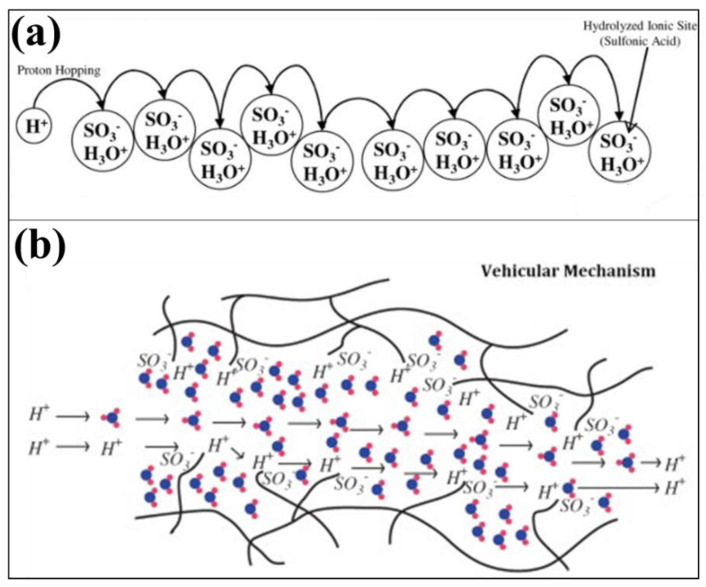
Diagram of (**a**) Grothus mechanism and (**b**) vehicular approach (permission to reproduce [49], License number: 5527551055459).

Figure 2a illustrates the process of hydrogen gas oxidation at the anodic electrode, particularly within the catalyst layer. During this process, electrons and protons are generated. By connecting an external circuit to the cell, the electrons can easily flow from the anode to the cathode. At the cathode, the electrons combine with oxygen gas and the protons to produce water. The classification of FCs is primarily determined by the type of membrane used in the development of the cell, as well as its operational range [23]. The membrane plays a crucial role in facilitating the movement of ions between the anode and cathode, thereby enabling the electrochemical reactions within the FC. Different types of membranes offer various characteristics and suitability for different applications, leading to the classification of FCs based on their specific membrane technologies. FCs encompass a variety of types, including high-temperature PEM-FCs [24,25], direct methanol FCs [26,27], solid oxide FCs [28,29], alkaline FCs [30,31], microbial FCs [32,33], direct ethanol FCs [34,35], phosphoric acid FCs [36,37], molten carbonate FCs [38], regenerative FCs [39,40], direct alcohol FCs [41,42], enzymatic FCs [43,44], direct ethylene glycol FCs [45], direct carbon FCs [46,47], and more. PEM-FCs are particularly preferred in the automotive sector due to the characteristics of the membrane used in their construction. Polymer electrolyte membranes (PEM) employed in PEM-FCs offer several advantages. They facilitate higher FC performance by enabling efficient ionic transport, minimizing hydrogen crossover, and serving as a barrier between the anode and cathode, allowing for the movement of electrons through the external circuit. The PEM also acts as a support for the electrocatalyst, which enhances the chemical reaction process [48]. Positioned between the anodic and cathodic electrodes, the PEM facilitates the transport of protons between the electrodes. Cation exchange membranes have been found to be particularly effective in ion transport. Figure 3 depicts two mechanisms by which protons move through the membrane: the Grothus mechanism and the vehicular mechanism. These mechanisms illustrate the pathways through which ions are transported within the PEM, contributing to the overall functionality of the FC system.

**Figure 4:**
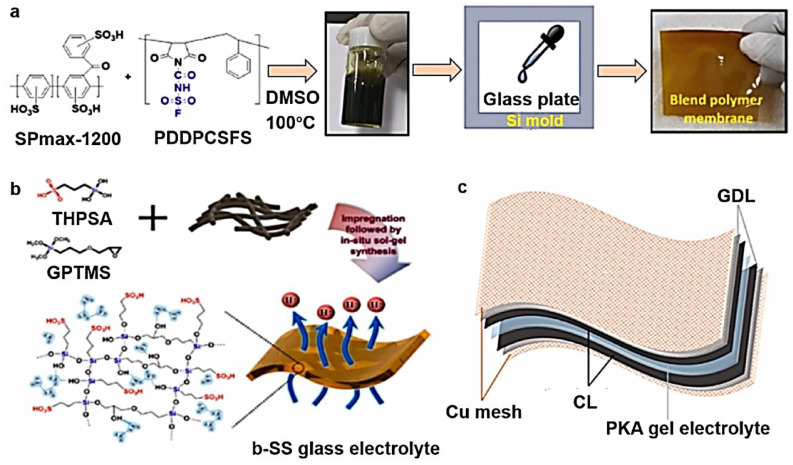
(**a**) Approach in fabricating blend polymer electrolyte. (**b**) Glass membrane approach and structural composition, and (**c**) structure of the developed membrane (permission to reproduce [55], License number: 5527711104310).

In low-temperature PEM-FCs, the electrolytes play a crucial role and are expected to exhibit specific characteristics. These include good proton conductivity, the effective suppression of hydrogen crossover, thermal and mechanical stability, and cost-effectiveness [50]. Ideally, low-temperature FCs operate at around 100 °C. Most electrolytes for these FCs consist of -SO_3_H groups. Among the various polymeric materials, perfluorosulfonic acid (PFSA) polymer membranes, particularly Nafion, have been extensively used due to their stability and high protonic conductivity [51]. Currently, there are several types of PFSA membranes available, distinguished by the length of the side chains. In addition to the widely used Nafion, other PFSA membranes include Aquivion^®^, Asiplex, Neosepta-F Flemion, and 3M ionomer [52]. These membranes offer alternative options with varying properties, allowing for customization based on specific application requirements. Despite the merits of PFSA-type membranes, such as Nafion, there are limitations associated with their manufacturing process, resulting in high costs. Additionally, these membranes exhibit a reduced performance under low humidity conditions [53]. To overcome these limitations, researchers have explored new types of membranes that can maintain cell performance at elevated temperatures. One such example is polybenzimidazole (PBI) membranes, which offer improved thermal and chemical characteristics. In addition to PBI membranes, other novel membranes have been synthesized with superior ion exchange characteristics and water absorption at 80 °C, as shown in Figure 4a [54]. The PBI membranes must be acid-doped to become a PEM, typically with phosphoric acid. These membranes demonstrated enhanced properties compared to the commercial Nafion 117 membranes. Beyond polymer-based membranes, there are silicate-based thin films that have shown promise for hydroxonium ion transport, making them suitable for use as PEMs. However, their application in FCs is limited due to their high cost and brittleness [55]. Other researchers have introduced polyimide nonwoven fabrics to enhance the characteristics of silicate-based PEMs. These PEMs were treated with 3-glycidylox-ypropyl trimethoxysilane (GPTMS), followed by sol–gel synthesis to create sulfonic acid functionalized silicate structures [56]. This approach results in PEMs with improved mechanical properties and enhanced cell performance, even under lower humidity and temperature conditions. The ratio of GPTMS used directly impacts proton conductivity through the membranes. Another novel type of membrane is the quasi-solid kalium polyacrylate hydrogel membrane [57]. This flexible membrane is particularly beneficial in direct methanol fuel cells (DMFCs), as it effectively prevents methanol penetration, enhancing the overall flexibility and performance of the FC. However, there are concerns about the mass production and performance issues associated with these novel membranes, particularly in PEM-FCs.

### 1.2. Research Gap, Objectives, and Originality

While previous research by Hamidi et al. [58] placed the foundation for investigating the performance of the Nafion 112 membrane in low-temperature FCs, there is still a research gap regarding the utilization of advanced techniques to optimize the power density of the FC. The current study aims to address this gap by employing ANFIS and the Salp swarm algorithm (SSA) to enhance the performance of the FC. The main objective of this study is to maximize the output power density of PEM-FCs. The specific objectives include:

- Developing an ANFIS model based on empirical data to simulate the output power density of the PEM-FC.
- Applying the SSA to identify optimal values for the input control parameters.
- Treating the three input control parameters of the PEM-FC as decision variables during the optimization process.
- Maximizing the output power density of the PEM-FC.

The originality of this research lies in the utilization of ANFIS and the SSA to optimize the power density of PEM-FCs. By employing these advanced techniques, the study aims to enhance the FC’s performance and contribute to the existing body of knowledge in the field. Additionally, the comparison of the results obtained from different optimization algorithms (PSO, EO, and GWO) adds originality to the study by providing insights into the effectiveness of the SSA for maximizing power density.

## 2. Experimental Approach

The experimental data gathered [54] focused on evaluating the performance of PEM-FCs under varying conditions, including reactant pressure, electrolyte compression percentage, and humidity. The membrane electrode assembly structure for the Nafion 112 membrane consists of the anodic and cathodic regions, along with the electrolyte. For this specific experiment, the anodic region comprised carbon paper Ballard, 20% platinum-carbon catalyst, 27% weight Nafion solution, alcohol (80 mL), water (20 mL), and a platinum loading of 0.39 mg.cm^−2^. The cathodic region included carbon paper Ballard, 20% platinum–carbon catalyst, 25% weight Nafion solution, alcohol (80 mL), water (20 mL), and a platinum loading of 0.39 mg.cm^−2^. The operating temperature of the FC was set at 75 °C, with the anode temperature measured at 80 °C. The experimental procedure involved several stages of voltage application. It began with applying a constant voltage of 0.6 V for 1800 s, followed by 0.2 V for 600 s and 0.7 V for 60 s. Subsequently, a constant voltage of 0.6 V was maintained for 3600 s, and then 0.5 V for 2400 s. Additional details regarding the experimental procedure can be found in [54]. The study investigated the impact of varying the percentage of the Nafion membrane and its correlation with relative humidity at different levels of membrane compression. It is worth noting that the “percentage of Nafion membrane” refers to the proportion or concentration of Nafion material within the membrane’s composition. In the context of FC technology and PEM-FCs, Nafion is a commonly used polymer electrolyte material known for its excellent proton conductivity. The percentage of Nafion in the membrane composition indicates how much of the membrane’s structure is made up of Nafion polymer. This parameter can have a significant impact on the performance and properties of the membrane, as it influences factors such as ion conductivity, mechanical strength, and water retention. In experiments or studies related to PEM fuel cells, researchers often vary the percentage of Nafion in the membrane to investigate how changes in its concentration affect the fuel cell’s performance. This variation allows for a better understanding of how different membrane compositions can impact factors such as proton conductivity, water management, and overall efficiency in the fuel cell system. Typically, the percentage of Nafion is controlled by adjusting the formulation of the membrane material during its fabrication process. Researchers may change the concentration of Nafion and other additives to optimize the membrane’s properties for specific applications, such as improving fuel cell performance under varying operating conditions. For more comprehensive information on the experimental procedure, refer to [54]. 

## 3. Methodology

The methodology employed in this study consists of two levels: ANFIS modeling and parameter identification through modern optimization techniques.

### 3.1. ANFIS Modeling

The ANFIS system, which stands for Adaptive Neuro-Fuzzy Inference System, employs membership functions within the fuzzification layer to establish a nonlinear mapping of inputs. This mapping process generates fuzzy rules in the inference engine phase. The outputs of these rules are evaluated, and the activated rules are aggregated in the normalized layer to produce the final fuzzy output. This fuzzy output is then converted from its fuzzy representation to a crisp value in the defuzzification layer. Gaussian-shaped membership functions and weighted-average defuzzification are commonly utilized in this process [59]. The ANFIS model is governed by IF-THEN rules, which define the input–output mapping. These rules represent the logical relationships between the input variables and the resulting output. An example of ANFIS rules is provided below:

IF *x* is *A*_1_ and *y* is *B*_1_ then *f*_1_ = *g*_1_(*x*, *y*) (1)

IF *x* is *A*_2_ and *y* is *B*_2_ then *f*_2_= *g*_2_(*x*, *y*) (2)

where the As and Bs are the membership functions of the two inputs *x* and *y*, respectively. However, the final output *f* is calculated based on the two rules’ outputs, *f*_1_ and *f*_2_, as follows:

(3) $$f={\tilde{\omega }}_{1}f_{1}+{\tilde{\omega }}_{2}f_{2}\;(\mathrm{Output}\mathrm{Layer})$$

(4) $$\mathrm{Evaluating}\;{\tilde{\omega }}_{1}g_{1}(x,y)\;and\;{\tilde{\omega }}_{2}g_{2}(x,y)\;(\mathrm{Defuzzification}\mathrm{Layer})$$

(5) $${\tilde{\omega }}_{1}=\frac{\omega_{1}}{\omega_{1}+\omega_{2}}\;\mathrm{and}\;{\tilde{\omega }}_{2}=\frac{\omega_{2}}{\omega_{1}+\omega_{2}}\;(N\mathrm{Layer})$$

(6) $$\omega_{1}=\mu_{A_{1}}*\mu_{B_{1}}and\;\omega_{2}=\mu_{A_{2}}*\mu_{B_{2}}(\pi \mathrm{Layer})$$

$$\mu_{A_{1}},\;\mu_{A_{2}},\mu_{B_{1}}\;\mathrm{and}\;\mu_{B_{2}}\mathrm{are}\mathrm{the}\mathrm{MF}\mathrm{values}\mathrm{of}\mathrm{the}\mathrm{two}\mathrm{inputs}\;(\mathrm{Fuzzification}\mathrm{Layer})$$

### 3.2. Salp Swarm Algorithm (SSA)

**Figure 5:**
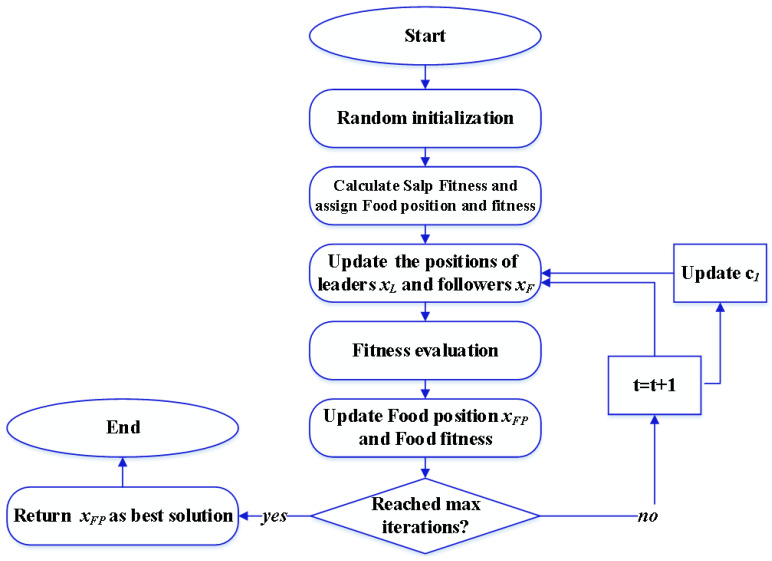
SSA flowchart.

The SSA, developed by Mirjalili et al. [60], is a nature-inspired metaheuristic optimization algorithm (MOA). It takes inspiration from the distinctive behavior of salp swarms, which organize themselves into long chains led by a leader. Since its introduction, SSA has demonstrated effectiveness in various applications. In the optimization process of SSA, the salps form a chain where one salp serves as the leader, while the others, called followers, synchronize their movements with the preceding salp’s location. The leaders, or first salps, adjust their positions in response to the location of food sources or desired objectives. The updating equation for SSA can be described as follows:

(7) $$\begin{array}{l} x_{L}(t+1)=\left\{ \begin{array}{l} \begin{array}{cc} x_{FP}(t)+c_{1}((ub-lb)c_{2}+lb) & c_{3}<0.5 \end{array}\\ \begin{array}{cc} x_{FP}(t)-c_{1}((ub-lb)c_{2}+lb) & c_{3}>0.5 \end{array} \end{array}\right.\\ x_{F}^{i}(t+1)=0.5(x_{F}^{i}(t)+x_{F}^{i-1}(t)) \end{array}$$

where *x_L_*, *x_F_*, and *x_FP_* indicate the leaders’, followers’, and food’s positions, respectively; *i* denotes the *i*th salp, *c*_1_ is a decay factor, and *c*_2_ and *c*_3_ are random numbers. The SSA flowchart is presented in Figure 5.

## 4. Results and Discussion

### 4.1. Modeling Phase

The construction of the ANFIS model involved the utilization of a dataset comprising 36 data points, which were split into two sets: a training set consisting of 28 points and a testing set for the remaining data points. To train the model, a hybrid approach combining the least squares estimation (LSE) method in the forward path and the backpropagation algorithm in the backward path was employed. The subtractive clustering (SC) technique was employed to generate the system’s rules, resulting in a total of 26 rules for this study. The models were trained iteratively until a lower root mean square error (RMSE) value was achieved, indicating improved performance and accuracy. The statistical metrics derived from the trained ANFIS model, including RMSE and other relevant indicators, are presented in Table 1. These metrics provide an assessment of the model’s predictive capability and its overall effectiveness.

**Figure 6:**
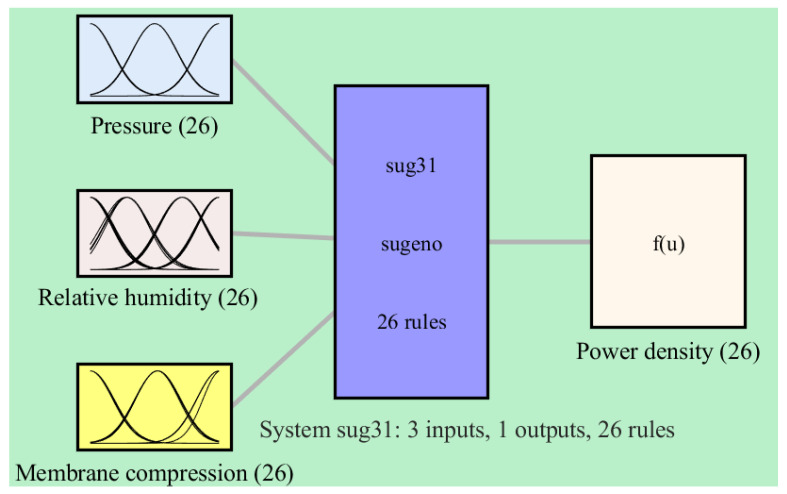
Configuration of fuzzy-based model of power of PEM-FC.

**Figure 7:**
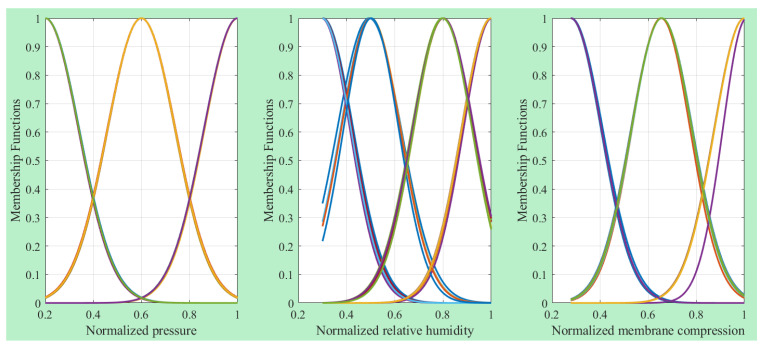
Inputs’ MFs of ANFIS model.

Table 1 presents the evaluation results of the ANFIS model for power density. The RMSE values obtained for the training and testing data are 0.0003 and 24.5, respectively. The coefficient of determination (R-squared) values are 1.0 for the training data and 0.9598 for the testing data. Overall, the coefficient of determination for all the data is 0.9914, indicating a successful modeling phase. Figure 6 depicts the construction of the ANFIS model, which consists of three input variables and a single output variable. This figure provides an overview of the model’s architecture, showcasing the input–output relationship captured by the ANFIS model. Additionally, Figure 7 illustrates the outlines of the Gaussian-shaped membership functions used in the ANFIS model. These membership functions define the fuzzy sets and their associated linguistic labels, which play a crucial role in the inference process of the ANFIS model.

**Figure 8:**
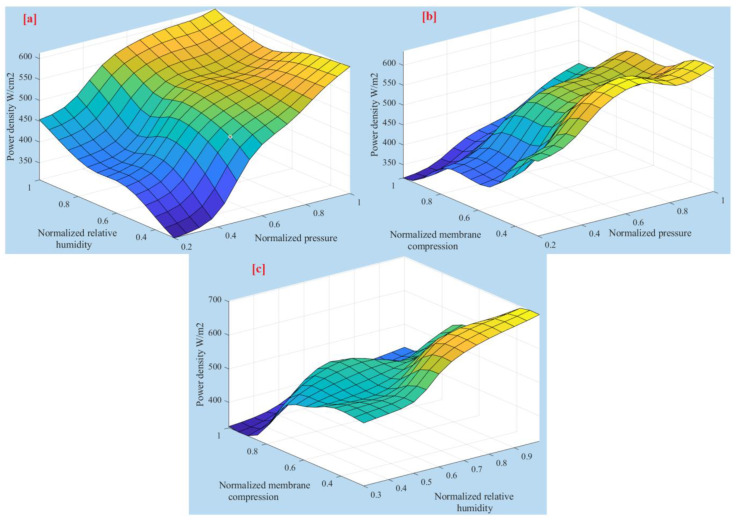
Three-dimensional plots of controlling parameters. (**a**) normalized relative humidity against normalized pressure (**b**) normalized membrane compression against normalized pressure and (**c**) normalized membrane compression against normalized relative humidity.

Figure 8 offers a three-dimensional perspective, illustrating the contours of the input–output function of the system. The figure showcases the relationship between the system’s inputs and the corresponding output by displaying the contours for each combination of two inputs at a time.

**Figure 9:**
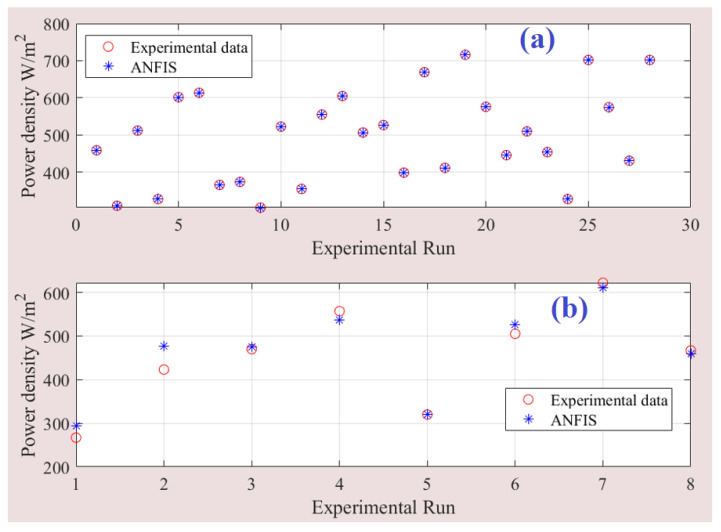
Predicted versus experimental data of ANFIS model: (**a**) training data, and (**b**) testing data.

**Figure 10:**
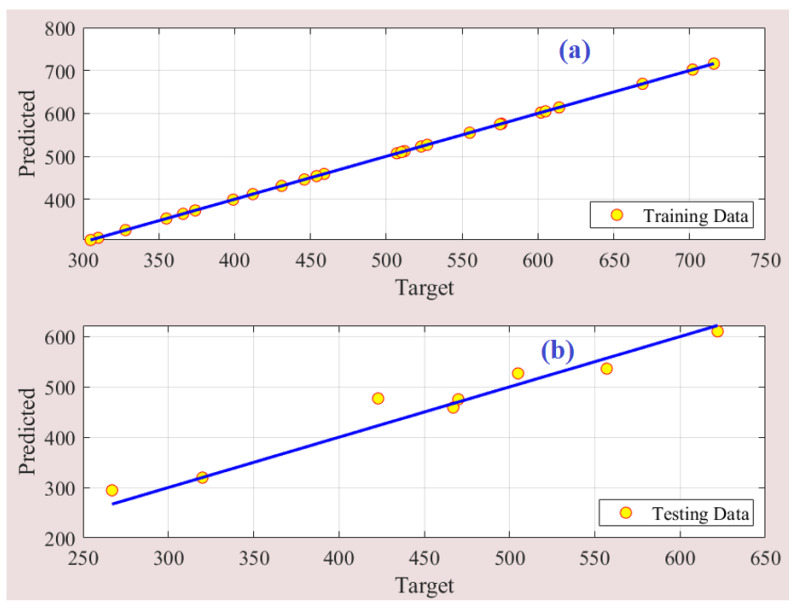
Prediction accuracy of fuzzy model. (**a**) training data, and (**b**) testing data.

The developed ANFIS model effectively captures the intricate relationship between the input and output parameters of the PEM-FC, resulting in accurate predictions of the output power density. This is evident from the comparison between the fuzzy model’s predicted outputs and the corresponding experimental data, as depicted in Figure 9. The plot clearly shows a close alignment between the estimated and measured data points, indicating a high degree of agreement and validation of the ANFIS model. To further evaluate the performance of the model, Figure 10 illustrates the predictions plotted around the one-hundred percent precision line for both the training and testing stages. The data points clustered around this line indicate a high level of precision and accuracy in the model’s predictions. 

### 4.2. Parameter Identification Process 

The optimization algorithm can play a significant role in improving the power density of PEM-FCs in practical applications. Its primary function is to provide a precise assessment of PEM-FC performance, assessing their suitability for specific applications, and identifying any limitations. In practical terms, this algorithm functions as a diagnostic tool, pinpointing specific deficiencies within the cell. The objective of this section is to optimize the output power of the PEM-FC by identifying the optimal values for the control parameters of pressure, relative humidity, and membrane compression. To achieve this, we first constructed a robust ANFIS model that accurately captures the relationship between the input parameters and the output power. Subsequently, we utilized SSA to solve the optimization problem and determine the optimal values for the control parameters. It is important to highlight that Yan et al. [61] tackled crucial factors, including flooding and membrane drying, which play a pivotal role in improving PEM-FC performance. In this study, the problem statement for the objective function can be summarized as follows:

(8) $$x=\underset{x\in R}{\arg}\max\;(y)$$

where *x* is the three controlling input variables and *y* is the power density of the PEM-FC.

Table 2 presents the optimal parameters and the corresponding maximum power density of a PEM-FC, as determined through experimental data and the SSA. The results demonstrate a notable agreement between the experimental data and the suggested approach.

**Figure 11:**
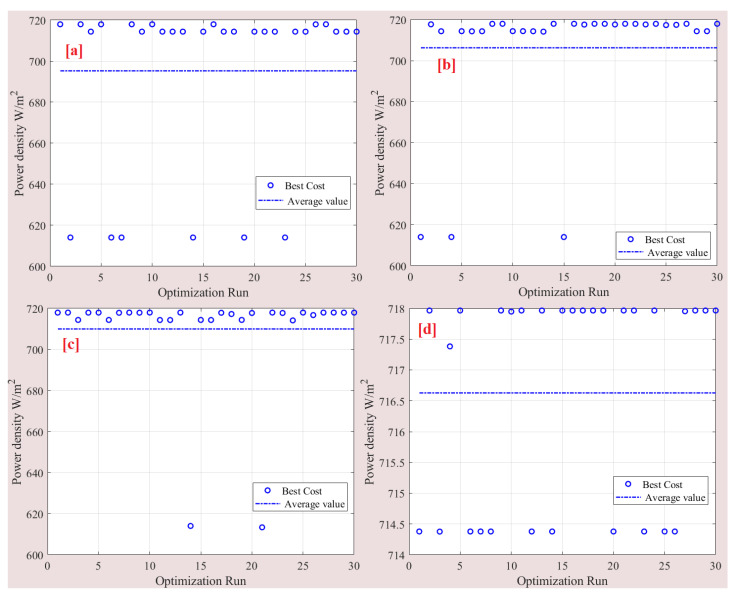
Detail of 30 runs: (**a**) PSO, (**b**) EO, (**c**) GWO, and (**d**) SSA.

To evaluate the effectiveness of the SSA compared to other optimization algorithms, namely particle swarm optimization (PSO), evolutionary optimization (EO), and grey wolf optimizer (GWO), we conducted a comparative analysis. To ensure a fair comparison, we maintained a consistent number of particles (5) and a maximum number of iterations (50) across all optimizers. To eliminate the influence of random results, each optimizer was run 30 times. The statistical analysis of these 30 runs is presented in Table 3. The results demonstrate that SSA outperformed the other algorithms in terms of average power density, achieving a value of 716.63 mW/cm^2^. GWO is closely followed with an average power density of 709.95 mW/cm^2^. In contrast, PSO exhibited the lowest average power density, measuring only 695.27 mW/cm^2^. The standard deviation values, which indicate the variability of the results, ranged from 1.72 to 40.63. Both SSA and GWO exhibited lower standard deviations, with values of 25.77, while PSO demonstrated a higher standard deviation of 40.63. For more detailed information about the cost function values and optimal parameters obtained in each run, refer to Table 4 and Figure 11. These resources provide a comprehensive overview of the optimization process and further support the superior performance of SSA in achieving higher power density values.

**Figure 12:**
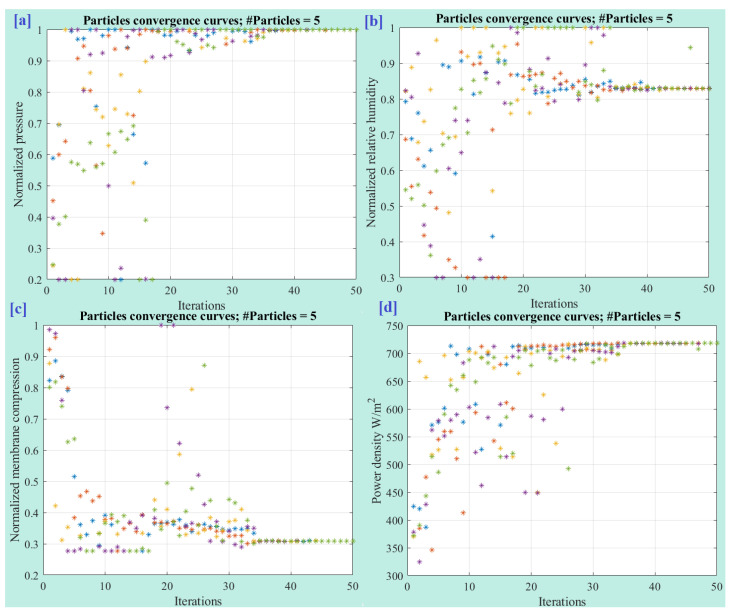
Particle convergence during the optimization process: (**a**) normalized pressure, (**b**) normalized relative humidity, (**c**) normalized membrane compression, and (**d**) power density.

**Figure 13:**
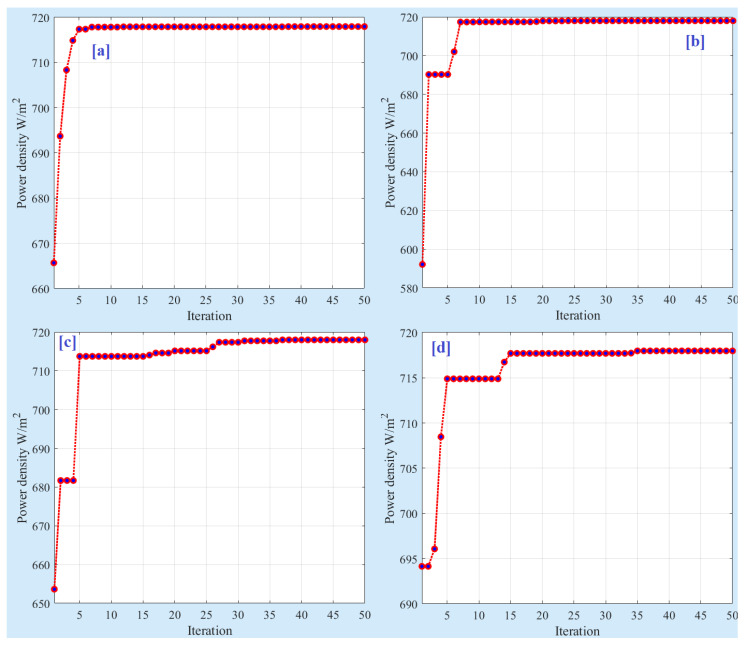
Best cost function variation for (**a**) PSO, (**b**) EO, (**c**) GWO, and (**d**) SSA.

Figure 12 displays the convergence of particles throughout the optimization process. The optimal values obtained for the normalized pressure (Figure 12a), normalized relative humidity (Figure 12b), and normalized membrane compression (Figure 12c) are 1.0, 0.82, and 0.308, respectively. These values represent the optimal settings for each parameter that lead to the highest power density of the FC. Furthermore, Figure 13 showcases the variation in the best cost function during the optimization process. The plot demonstrates how the cost function, which represents the objective to maximize the output power density, evolves over the iterations. 

**Figure 14:**
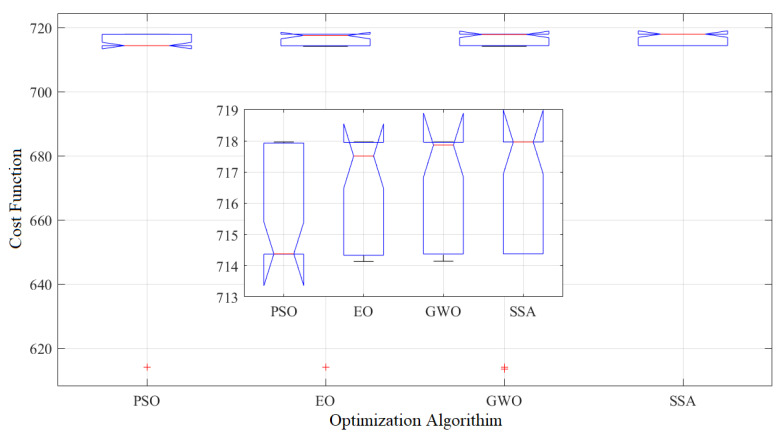
ANOVA ranking.

To further validate the obtained results and solidify the findings, two additional tests, namely ANOVA (Analysis of Variance) and Tukey tests, were conducted. Table 5 presents the results of the ANOVA test, which confirms the significant differences in the outcomes between the various optimization algorithms. The test examines the variations in the performance of the algorithms and provides statistical evidence of the superiority of one algorithm over the others. Figure 14 illustrates the ANOVA ranking, which highlights the performance of each algorithm based on mean fitness and variations. The ranking confirms that the SSA exhibits superior performance compared to the other algorithms, as it yields higher mean fitness values and variations.

**Figure 15:**
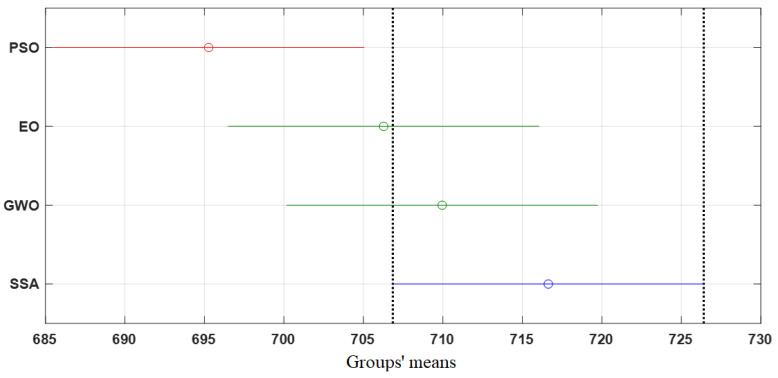
Tukey test.

The Tukey test was conducted to validate the results obtained from the ANOVA analysis, providing further insights into the differences among the optimization algorithms. Figure 15 presents the results of the Tukey test, which reveals significant differences in the mean values between the various groups. Specifically, the mean of the PSO group is significantly different from the SSA group. This indicates that the performance of PSO differs significantly from that of SSA. Moreover, the EO and GWO groups exhibited relatively good performance compared to the SSA group.

**Figure 16:**
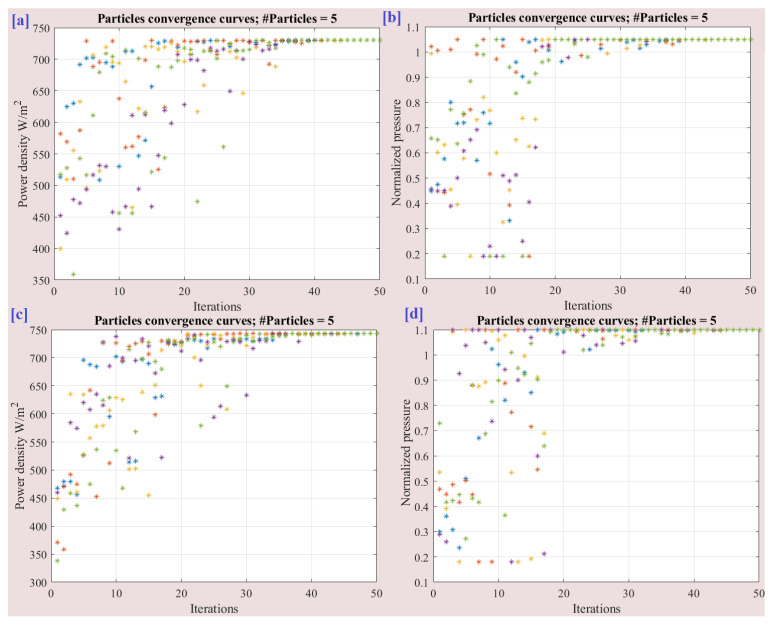
Particle convergence with the parameter extension. (**a**) Power density with 5% extension, (**b**) normalized pressure with 5% extension, (**c**) Power density with 10% extension and (**d**) normalized pressure with 10% extension.

The optimal input parameters for pressure, relative humidity, and membrane compression were determined to be 25, 82, and 5.544, respectively, as previously mentioned. In order to further explore the impact of these parameters, an extension of 5% and 10% were tested for the upper and lower values. Table 6 presents the optimized results with the extended input parameters. It can be observed that the optimal values for relative humidity and membrane compression remained unchanged at 82 and 5.544, respectively, indicating that the proposed extensions had little to no effect on these parameters. However, the pressure value showed an increase with the increase in the extension percentage. For instance, with a 5% extension, the power density improved from 717.96 mW/cm^2^ to 730.27 mW/cm^2^, representing an enhancement of approximately 1.715%. Figure 16 illustrates the convergence of particles with the parameter extension. It provides a visual representation of how the optimization algorithm adapted to the extended input parameters during the optimization process. The convergence of the particles indicates the algorithm’s ability to find improved solutions as it progresses.

## 5. Conclusions

This study aimed to enhance the output power of PEM-FCs through the use of the ANFIS and optimization algorithms. The study successfully developed a robust ANFIS model based on experimental data, which accurately simulated the FC’s output power density considering the parameters of pressure, relative humidity, and membrane compression. To determine the optimal values of the input parameters, the SSA was employed. During the optimization process, the three input parameters of the PEM-FC were treated as decision variables, and the cost function was used to maximize the output power density of the PEM-FC. The modeling stage demonstrated the effectiveness of the ANFIS model, with RMSE values of 0.0003 and 24.5 for training and testing data, respectively. The coefficient of determination values further confirmed the successful modeling, with values of 1.0 and 0.9598 for training and testing, respectively. To ensure the reliability of SSA, the results were compared with other optimization algorithms, including PSO, EO, and GWO. Among these algorithms, SSA exhibited the highest average power density of 716.63 mW/cm^2^, followed closely by GWO at 709.95 mW/cm^2^. PSO yielded the lowest average power density of 695.27 mW/cm^2^. These findings highlight the effectiveness of the ANFIS model and the superiority of SSA in optimizing the output power density of PEM-FCs. The results contribute to the advancement of clean energy generation by providing valuable insights into the optimal parameters for maximizing the performance of FCs.

## Figures and Tables

**Table 1:** RMSE and coefficient of determination values of ANFIS model of power density.

RMSE			Coefficient of Determination (*R*^2^)		
Train	Test	All	Train	Test	All
0.0003	24.5	11.5535	1.0	0.9598	0.9914

**Table 2:** Optimal parameter values using measured data and proposed strategy.

Method	Pressure	Relative Humidity	Membrane Compression	Power Density mW/cm^2^
Measured	25	80	5	716
ANFIS and SSA	1.0 (N *)	0.82 (N *)	0.308 (N *)	717.96
	25	82	5.544	

* N means normalized.

**Table 3:** Statistical evaluation of PSO, EO, GWO, and SSA.

	PSO	EO	GWO	SSA
Maximum	717.97	717.97	717.97	717.97
Minimum	614.05	614.04	613.39	714.38
Average	695.27	706.27	709.95	716.63
STD	40.63	30.78	25.77	1.72
median	714.38	717.51	717.87	717.97
variance	1651.05	947.67	663.86	2.94

**Table 4:** Detail of 30 runs.

Run	PSO	EO	GWO	SSA	Run	PSO	EO	GWO	SSA
1	717.96	614.06	717.94	714.38	16	717.96	717.96	714.34	717.97
2	614.06	717.7	717.95	717.97	17	714.38	717.58	717.87	717.97
3	717.97	714.33	714.38	714.38	18	714.38	717.96	717.26	717.97
4	714.38	614.04	717.93	717.38	19	614.06	717.95	714.38	717.97
5	717.95	714.36	717.95	717.97	20	714.38	717.63	717.76	714.38
6	614.05	714.3	714.37	714.38	21	714.38	717.96	613.39	717.97
7	614.06	714.33	717.87	714.38	22	714.37	717.93	717.96	717.97
8	717.96	717.94	717.96	714.38	23	614.06	717.65	717.78	714.38
9	714.38	717.97	717.9	717.97	24	714.38	717.94	714.14	717.97
10	717.97	714.38	717.97	717.95	25	714.37	717.33	717.97	714.38
11	714.38	714.37	714.34	717.97	26	717.93	717.45	716.73	714.38
12	714.38	714.33	714.33	714.38	27	717.97	717.96	717.95	717.95
13	714.38	714.14	717.96	717.97	28	714.38	714.34	717.97	717.97
14	614.06	717.96	614.06	714.38	29	714.38	714.35	717.94	717.97
15	714.37	614.04	714.37	717.97	30	714.38	717.96	717.94	717.97

**Table 5:** ANOVA results.

Source	df	SS	MS	F	Prob
Columns	3	7188.6	2396.2	2.84	0.014
Error	116	97,965.7	844.5		
Total	119	105,154.3			

**Table 6:** Optimized results with extension of the input parameters.

Method	Pressure	Relative Humidity	Membrane Compression	Power Density W/cm^2^
5%	1.05	0.82 (N *)	0.308 (N *)	730.27
	26.5	82	5.544	
10%	1.1	0.82 (N *)	0.308 (N *)	742.93
	27.5	82	5.544	

* N means normalized.

## Data Availability

Data will be made available upon request.
